# A glimpse into the molecular mechanism of integral membrane proteins through hydrogen–deuterium exchange mass spectrometry

**DOI:** 10.1002/pro.3853

**Published:** 2020-03-25

**Authors:** Chloe Martens, Argyris Politis

**Affiliations:** ^1^ Laboratory for the Structure and Function of Biological Membranes, Center for Structural Biology and Bioinformatics Université Libre de Bruxelles Brussels Belgium; ^2^ Department of Chemistry King's College London London UK

**Keywords:** allosteric coupling, conformational dynamics, GPCRs, hydrogen–deuterium exchange mass spectrometry, integral membrane proteins, transporters

## Abstract

Integral membrane proteins (IMPs) control countless fundamental biological processes and constitute the majority of drug targets. For this reason, uncovering their molecular mechanism of action has long been an intense field of research. They are, however, notoriously difficult to work with, mainly due to their localization within the heterogeneous of environment of the biological membrane and the instability once extracted from the lipid bilayer. High‐resolution structures have unveiled many mechanistic aspects of IMPs but also revealed that the elucidation of static pictures has limitations. Hydrogen–deuterium exchange coupled to mass spectrometry (HDX‐MS) has recently emerged as a powerful biophysical tool for interrogating the conformational dynamics of proteins and their interactions with ligands. Its versatility has proven particularly useful to reveal mechanistic aspects of challenging classes of proteins such as IMPs. This review recapitulates the accomplishments of HDX‐MS as it has matured into an essential tool for membrane protein structural biologists.

## 1 INTRODUCTION: STRUCTURAL BIOLOGY OF MEMBRANE PROTEINS

Structural biology provides invaluable insight into the mechanism of diverse biological macromolecules. Through the microscope or with the help of other biophysical tools, structural arrangements can be observed or deduced. This becomes a challenging task when the molecule of interest is embedded in a biological membrane. Structural biology of integral membrane proteins (IMPs) often implies extraction from the membrane, a process far from trivial.^1^ In order to keep the protein in its folded and functional state, the presence of a membrane surrogate is an absolute necessity for any structural investigation.

A detailed understanding of the molecular mechanism of any protein of interest is required to link structure to function. Deciphering the molecular mechanism requires the integration of structural information with dynamic information. In other words, one has to be able to monitor different motions the protein undergoes during a functional cycle, and to identify the allosteric networks associated with signal transmission. In that regard, HDX‐MS can provide invaluable information.^2^, ^3^ HDX‐MS reports on the exchange of labile hydrogens from the amide backbone of proteins with deuterium from the solvent. This exchange is directly related to solvent accessibility and stability of the H‐bond, and can be used to follow structural changes in proteins and their complexes.^4^

There has been a recent surge in the application of HDX‐MS to IMPs. This is particularly timely as it correlates with the massive increase in structures resolved by high‐resolution methods, such as cryogenic electron microscopy (Cryo‐EM) and X‐ray crystallography.^5^ Indeed, since IMPs are embedded in a membrane or a membrane surrogate, a significant portion of the protein will not be solvent accessible. Most of the time, only the extracellular membrane domains will undergo HDX and yield structural information. Within the structural framework provided by atomic resolution structures, structural dynamics of the solvent accessible domains can be linked with global dynamic changes and provide useful details on conformational dynamics of IMPs.

In this review, we will focus exclusively on efforts that employ HDX‐MS to the study of IMPs. Other recent reviews have wonderfully recapitulated the recent technical progresses made by the technique and the nuts and bolts of data interpretation, artefact detection, and prevention. These reviews also provide a great theoretical background of the fundamental principles of HDX which will not be covered here.^2^, ^6^, ^7^, ^8^ Section 1 will review the biological insights that were obtained through this technique, and specifically the research carried out on IMPs extracted from the membrane and solubilized within detergent micelles. The study of IMPs in conditions closer to the cell is the obvious next challenge. The significant steps toward that aim that have already been taken will be reviewed in Section 2. Along the way, the challenges associated with the handling of IMPs for HDX‐MS studies will also be discussed. Finding the right conditions for efficient digestion, working in the presence of detergents and/or lipids, accounting for the intrinsic heterogeneity of membrane proteins‐lipid complexes are different hurdles that are slowly being overcome with the help of better chromatographic and MS systems and data analysis workflows (Table 1).

**Table 1:** Summary of the different parameters that can be modulated to improve HDX‐MS analysis

Modulation of experimental conditions for improved sequence coverage and data quality	
Quench and digestion	*Standard*: pH: 2.5 Temperature: 0°C Protease: Porc pepsin *√ Other proteases*: Rhizopuspepsin^9^ Nepethesin I Nepenthesin II^10^ *√ On‐column* digestion yields more peptides than in‐solution digestion.^11^ It is worth making its own column with its favorite protease.^12^ *√ Increased pressure* on‐column by increasing flow^13^ or using a back‐pressure regulator.^14^ So far only possible on BEH pepsin columns. *√ Increased protein concentration* 15, 16 *√ Useful additives*: Urea, TCEP, Gu‐HCl,^13^ and detergent
Liquid chromatography‐mass spectrometry	*Standard*: *LC*: Reverse‐phase chromatography on C18 column with prior trapping for desalting. *MS*: Often Synapt or Xevo—G2 in MS^E^ mode **√** Drift‐time aligned MS^E^ (HDMS^E^)^17^ **√** LC column with shorter alkyl chain—for example, BEH C4 or C8^13^ **√** Gradient optimization (8–30%, 8–50%)^15^ **√** Saw‐tooth gradient after peptides elution to prevent carryover^16^
Miscellaneous observations	Use of PEG‐based detergents (e.g., Triton X‐100) complicates peptides identification. Good practice to start with a benchmark condition—for example, locked protein.^16^, ^18^, ^19^ Adding TCEP helps but too much (>1 M) reduces spectral quality.^15^

*Note*: The tick indicates parameters that have shown improvement compared with the standard conditions.

Abbreviations: BEH, Ethylene Bridged Hybrid; HDX‐MS, hydrogen–deuterium exchange coupled to mass spectrometry; LC, Liquid Chromatography; MS, Mass spectrometry; PEG, Polyethylene Glycol; TCEP, tris(2‐carboxyethyl)phosphine; HDMS, High Definition MS .

A word of caution also accompanies this review. As the interest for the technique and its applications expands exponentially, we are constantly reminded that the fundamentals of HDX theory are still work in progress.^20^ As the complexity of the systems amenable to HDX‐MS increases, so is the difficulty in the data interpretation and analysis^21^ and the risk of generating artefacts.^22^ The recent growth of the HDX‐MS field has led to the publication of recommendations to report HDX‐MS data. Such white paper usually is a good indicator that a field has passed its adolescence and is now mature—but still relatively young.^23^

## 2 HDX‐MS STUDIES OF MEMBRANE PROTEINS IN DETERGENT MICELLES

Detergent micelles remain the gold standard of membrane mimics. The hydrophobic tail of the detergent molecule shields the hydrophobic region of the IMP and the polar region faces the solvent, thus preventing aggregation in solution. Despite differing notably from the biological membrane, detergent micelles allow the isolation of many IMPs in a stable and functional state. The majority of biochemical and biophysical studies of IMPs have been done in detergent micelles and have unveiled the inner workings of many proteins. Specifically, most high‐resolution structures of IMPs have been obtained in detergent micelles.

IMPs adopt different forms and functions but three major classes are overrepresented in terms of structural studies: transporters, channels and receptors. Two out of these three classes are also overrepresented in HDX‐MS research works: transporters and receptors. The average high‐molecular weight (~400 kDa or more) and oligomeric state of channels complicate data analysis and currently restricts their suitability for HDX‐MS studies, although it is likely that progresses in automatic data analysis will overcome this limitation.

### 2.1 *Transporters*

**Figure 1:**
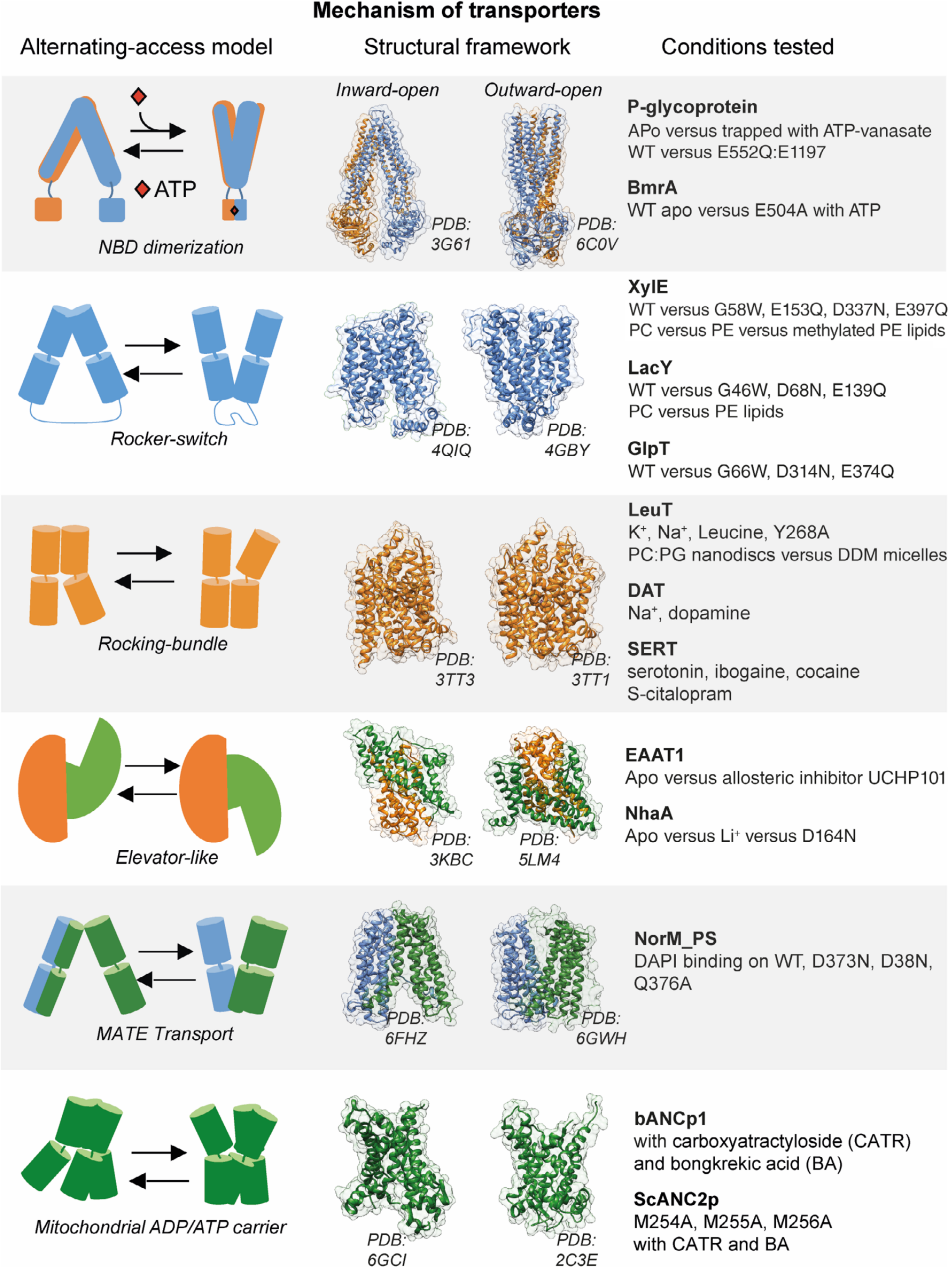
Example of transporters studied by HDX‐MS. The left panel shows alternating‐access models used by various transporters. The middle panel illustrates the IF and OF states with selected high‐resolution structures. The right panel summarizes the conditions tested by HDX‐MS to understand the role of specific parameters in the molecular mechanism of transport. HDX‐MS, hydrogen–deuterium exchange coupled to mass spectrometry; IF, inward facing; OF, outward facing

Transporters is the class of IMPs that has been the most studied by HDX‐MS (Figure 1). Their relatively small size (usually ≤100 kDa) and the availability of reliable purification protocols make them easily amenable to HDX‐MS. Furthermore, it is well established that transporters have to alternate between two major functional states open to opposite sides of the membrane, called inward‐facing (IF) and outward‐facing (OF) states^24^, ^25^, ^26^ (Figure 1). Solvent accessibility changes dramatically between both states, and this change can be measured by HDX‐MS.^4^ Thus HDX‐MS is well suited to follow conformational changes in transporters.

Transporters can be broadly divided into two main classes: ABC transporters that rely on energy input from ATP hydrolysis to transport their substrate and secondary transporters that harness the energy potential contained in a transmembrane (TM) gradient to power substrate translocation. ABC transporters possess a cytoplasmic region comprising two nucleotide binding domains (NBDs) where ATP binds and is hydrolyzed. The presence of an important solvent accessible region on the transporter also facilitates HDX‐MS studies of this type of IMPs.

#### 2.1.1 *ABC transporters*

The multidrug exporter BmrA was the first ABC transporter to be studied by HDX‐MS.^27^ This study demonstrated the usefulness of HDX‐MS to observe conformational transitions of transporters. The protein was locked in the closed/outward conformation with E504A mutation (a conserved catalytic residue adjacent to the walker B motif^28^) in the presence of ATP, and its deuteration behavior was compared with that of the wild‐type (WT) apo protein. Significantly different patterns of HDX allowed to distinguish between the closed state from the open state of the WT protein.

Similar strategies were used to follow the conformational transition of the eukaryotic ABC transporter P‐gP.^13^, ^29^ This transporter is a multidrug efflux pump, shown to play a role in the resistance phenotype of different cancer cells.^30^ The conformational dynamics were studied by two different research groups, aiming at understanding the coupling between ATP hydrolysis and conformational changes. Both studies looked at the differences between the apo form and the post‐hydrolysis state trapped with vanadate, which should reflect the OF conformation. Kopcho et al. managed to obtain a coverage of 85% of the sequence after extensive optimization (Table 1), compared with 40% obtained in the earlier work of Li et al. In both studies, it is clear that vanadate trapping causes a decrease in HDX in the NBDs, consistent with the dimerization of the domains. The improved coverage obtained by Kopcho et al. allows to link information from the NBDs with the extracellular domain. As expected, this region displays an increased HDX upon vanadate trapping, consistent with the increased flexibility and solvent accessibility of the OF conformation. Both studies report that the transporter shows hallmarks of a very complex conformational landscape and a dynamic binding pocket, which correlates with the substrate promiscuity of P‐gP. Kopcho et al. also compared the WT protein with a mutant mimicking the pre‐hydrolysis state (E552Q/E1197Q) and found that HDX is decreased in otherwise dynamic regions, leading them to suggest that the protein is in an occluded conformation.

It is interesting to note that all three studies highlighted asymmetry between the NBDs of the ABC transporters BmrA and P‐gP. These findings are in line with other spectroscopic studies using on P‐gP using DEER or single‐molecule FRET.^31^, ^32^

#### 2.1.2 *Neurotransmitter: Sodium symporters family*

LeuT is the prototypical protein from the neurotransmitter: sodium symporters (NSSs) family. This family of transporters has been under the spotlight for decades, because of their role in neurotransmission termination.^33^, ^34^ LeuT is an NSS homolog from the bacteria *Aquifex Aeolicus* and has long been used as a bacterial model of human homologs, before the recent structure resolution of the human NSS transporters DAT and SERT.^35^, ^36^ LeuT was studied by HDX‐MS in detergent micelles^37^ and nanodiscs.^18^, ^37^ In their work, Adhikary et al. used LeuT mutant Y268A to lock the transporter in the IF conformation, based on previous crystallographic work.^38^ In order to favor the OF conformation, they used saturating amount of sodium on the WT protein. They compared the HDX pattern between both states in order to identify the regions that undergo changes upon conformational transition, and observed that the region EL4a is a consistent reporter of the expected changes in solvent accessibility. Another study by Rand et al. investigated the role of ion and leucine binding on the conformational equilibrium and found the same hallmarks of the conformational transition. Both groups demonstrated the usefulness of HDX‐MS to study the role of conserved residues (Y268) and ligand binding (Na^+^ and leucine) on the conformational dynamics. Furthermore, Rand et al. identified specific motif unfolding, supporting the notion that a transient local unfolding can be necessary for a global conformational transition to happen.

The human NSS transporters DAT^39^ and SERT^40^ were recently studied as well. These transporters are known drug targets and a better understanding of their molecular mechanism has implications for drug addiction and depression therapies. These were one of the very few HDX‐MS studies carried out on transporters expressed and purified from mammalian cells, known to be more challenging to work with than their prokaryotic homologs. An extensive optimization of the digestion and labeling protocols were required which are summarized in a very useful methodological article.^11^ The DAT transporter was studied in the presence and absence of the Na^+^ and neurotransmitter dopamine. Specific locations were shown to behave differently upon Na^+^ and dopamine binding. Specifically, the intracellular loop (ICL) between TM8 and TM9 reports increased HDX upon sodium then dopamine binding. A thorough characterization of the conformational dynamics of SERT was carried out by the same research group. The effect of sodium, substrate serotonin (5‐HT), and inhibitors cocaine, citalopram, and ibogaine used as therapeutic or recreative drugs was systematically studied. This work revealed that EL4 and the T1a regions were consistent reporters of conformational changes, pointing them out as dynamic regions during the transport cycle. These regions present different patterns of HDX in the apo state, substrate (5‐HT)‐bound state, and inhibitor‐bound state. Interestingly, this study revealed opposite HDX patterns for the noncompetitive inhibitor ibogaine compared with competitive inhibitors cocaine and citalopram, indicating that a different mode of inhibition yields different structural dynamics.

#### 2.1.3 *Major facilitator superfamily*

**Figure 2:**
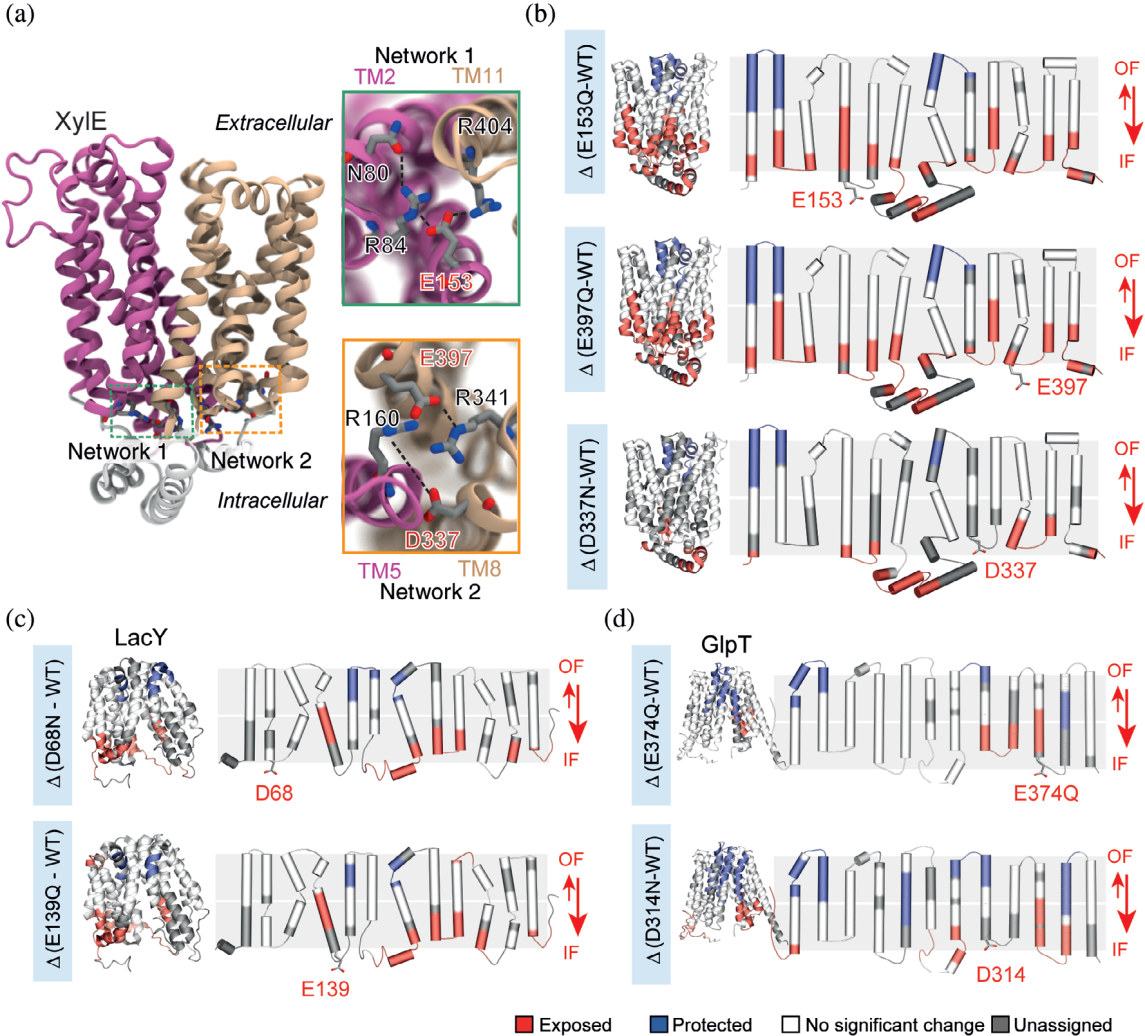
ΔHDX‐MS on MFS transporters LacY, XylE, and GlpT identifies a conserved mechanism of conformational cycling. (a) Two charge‐relay networks of conserved residues stabilize the OF state of XylE (PDB: 4GBY). Close‐up of networks 1 and 2—highlighted in green and orange, respectively—show the connection between the two lobes and highlight in red the mutated acidic residues. (b) ΔHDX between the mutants E153Q, E397Q, and D337N versus WT mapped onto the 3D and topological structure of XylE. (c) ΔHDX of the mutants D68N, and E139Q versus WT mapped onto the 3D and topological structure of LacY. (d) ΔHDX of the mutants E374Q and D314N mapped onto the 3D and topological structure of GlpT. Reproduced with permission from Reference 19 (distributed under CC‐BY license). HDX‐MS, hydrogen–deuterium exchange coupled to mass spectrometry; MFS, major facilitator superfamily; OF, outward facing; PDB, Protein Data Bank coordinates; WT, wild type

Proteins from the major facilitator superfamily (MFS) are ubiquitous—the overall architecture of 12 TM helices separated in two pseudo‐symmetric bundles is found in bacteria and humans, suggesting conserved mechanistic features.^41^ Three bacterial transporters LacY, XylE, and GlpT were studied in parallel in an extensive study aiming at identifying conserved motifs regulating the conformational transitions.^19^ This work introduces the use of a benchmarking strategy to identify peptides that can confidently and consistently be used as conformational reporters of the transition between IF and OF states, to help the downstream interpretation of data aimed at answering biological questions (Table 1). As an example, the authors replace a conserved glycine on TM2 by a bulky tryptophan in order to artificially lock the three transporters in the OF conformation. They carry out differential HDX‐MS experiments of the locked versus the WT transporters and obtain three ΔHDX patterns representative of the transition toward the OF conformation. They proceed to introduce conservative mutations of charged residues conserved throughout the MFS family and identify a charge‐relay network acting as a “general” conformational switch within the family (Figure 2). The authors combine the HDX‐MS studies with molecular dynamics (MD) simulations to reveal the role of such residues in the regulation of conformational transitions. This work reveals the efficiency of HDX‐MS to study homologs in parallel in order to identify conserved mechanisms. It showcases the synergy between MD and HDX‐MS where the predictions made by the first technique can be tested experimentally by the second.

#### 2.1.4 *Mitochondrial ADP/ATP carriers*

To the best of our knowledge, the first transporter studied by HDX‐MS was the bovine ADP/ATP carrier bANC1p from the mitochondria.^42^ This study made use of an automated solvent extraction technique integrated in an HPLC system to remove the detergent Triton‐X100 before peptides separation, and hence facilitate data analysis. The authors studied two different complexes of the bANC1p, one with the toxin carboxyatractyloside (CATR) and the other with the toxin bongkrekic acid (BA), which stabilize different functional states of the carrier.^43^ Clear changes in solvent accessibility allowed the authors to conclude that the CATR‐complex favors the conformation accessible to the intracellular matrix space, while the BA complex forms a peptide plug on the IMS side, a structural change coupled to an opening on the matrix side. In another study, the same team assessed the role of a conserved motif in the carrier family by looking into the yeast ADP/ATP carrier ScANC2p, this time using the detergent dodecyl‐maltoside which does not overlap with peptides chromatographic elution^44^ (Table 1). The authors could confirm the role of the BA and CART toxin, and further pinpoint the conserved mechanism of conformational transition mediated by the conserved ANCp family signature RRRMMM motif.

#### 2.1.5 *Excitatory amino acid transporter 1*

A very interesting study showed how the power of HDX‐MS can be harnessed to go beyond structural information and confirm a specific mechanistic hypothesis. The authors solved the structure of the human excitatory amino acid transporter EAAT1 by crystallography.^45^ The location of a noncompetitive inhibitor UCHP101 was resolved and the authors used HDX‐MS to reveal the mechanism of inhibition. The ΔHDX pattern obtained by comparing the protein with and without UCHP101 identified specific peptides displaying H/D decrease upon binding. The authors plotted the identified peptides on the structure crystallized the IF and on a model of the protein in the OF conformation. They could immediately deduce that inhibitor binding prompted a conformational transition and stabilization of the OF conformation, thus preventing conformational cycling supporting function.

#### 2.1.6 *Na^+^/H^+^ antiporter family*

Another pioneering study examined the conformational changes of the Na^+^/H^+^ exchanger NhaA. The structure of NhaA has been obtained at acidic pH where the protein is inactive, and its mode of alternating‐access is not established. HDX‐MS studies at physiological pH 7.5 allowed to monitor the structural rearrangements upon ion binding.^46^ The authors infer from the pattern of ΔHDX of the WT and a nonfunctional mutant D164N that the transporter is using an elevator‐like mechanism, wherein the immobile binding site is alternately shielded and exposed by the sliding of a transport domain.

#### 2.1.7 *Na^+^/Ca^2+^ exchanger family*

A study on the exchanger NCX_Mj responsible for Ca^2+^ homeostasis is an elegant demonstration that obtaining a lot of sequence coverage is not always necessary to answer specific mechanistic questions.^47^ NCX transporters exchange three Na^+^ ions for one Ca^2+^ but the binding sequence of events is difficult to understand. The crystal structure of NCX_Mj revealed four putative ion binding sites, three for Na^+^ binding sites and one for Ca^2+^.^48^ To confirm the location of specific ion binding, Giladi et al. performed HDX‐MS experiments in the presence and absence of Ca^2+^ and Na^+^. They obtain only 12 peptides but those included 10 of the 12 ion‐coordinating residues. By performing ΔHDX measurements, the authors could confirm that two ion binding sites are binding Na^+^, one is binding Ca^2+^ and another one is occupied by a water molecule, a finding in line with computational studies.^49^

#### 2.1.8 *Multidrug and toxin extrusion family*

The conformational dynamics of the multidrug transporter NorM_PS from the multidrug and toxin extrusion (MATE) superfamily were studied using HDX‐MS by Eisinger et al.^50^ Upon binding of the substrate DAPI, important structural changes were observed that confirmed the location of DAPI binding and also suggested a conformational transition toward an OF state. The role of specific conserved residues D373, D38, and Q276 in substrate binding was also tested and the authors could discriminate between residues involved in binding and those involved in allosteric structural changes.

In conclusion, all the above‐mentioned studies have showcased the value of HDX‐MS as a sensitive tool to follow conformational transitions of transporters in the context of the alternating‐access model. This technique can easily identify how ligand binding and conserved structural motifs control these transitions.

### 2.2 *G protein coupled receptors*

**Figure 3:**
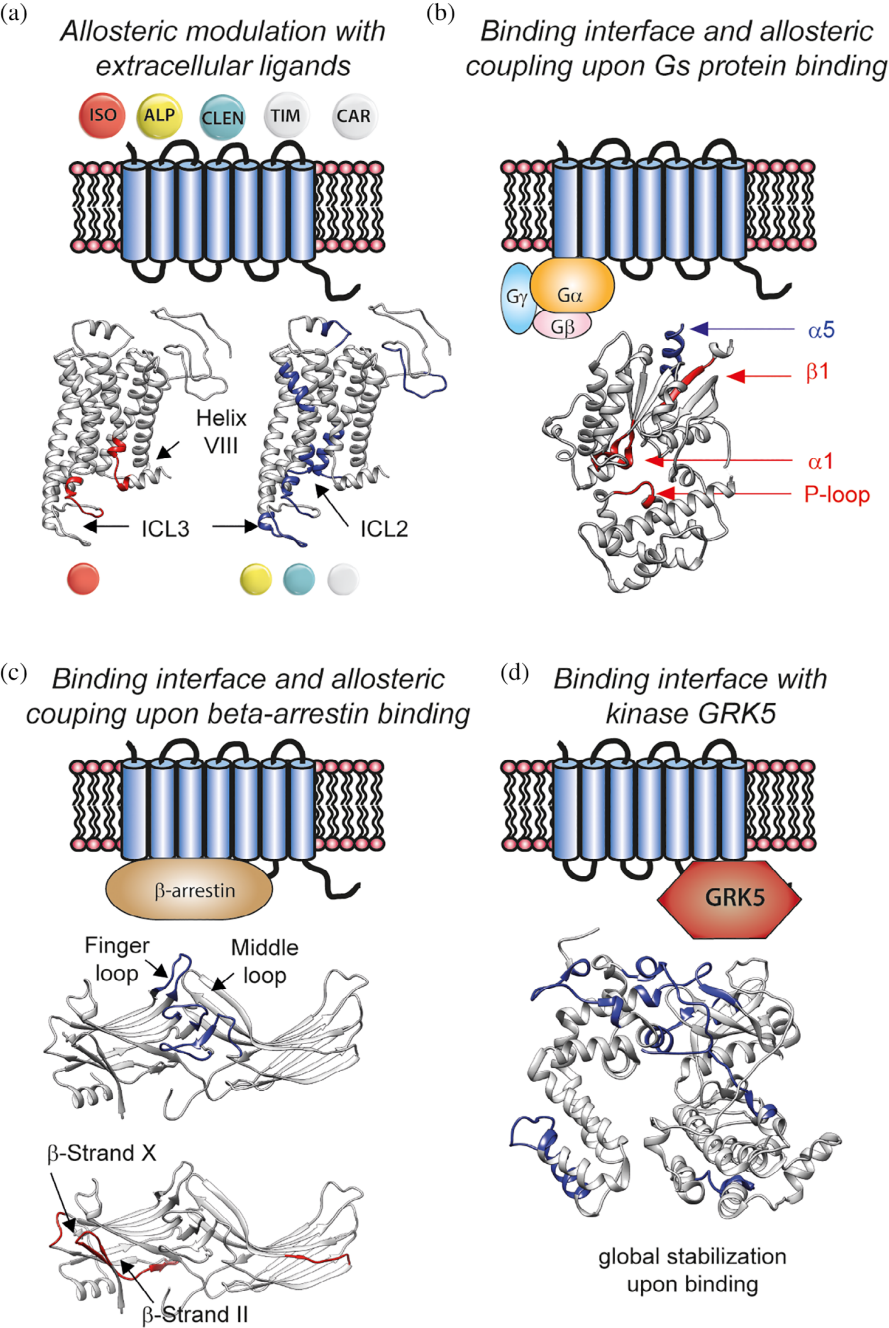
HDX‐MS studies on β2AR highlight distinct structural dynamics upon ligand and protein binding. (a) Differences between agonist isoproterenol and other ligands are clearly identified by HDX. (b) Helix α5 of the Gα subunit interacts with the receptor. Allosteric coupling causes destabilization of regions far from the binding interface. (c) Binding of β‐arrestin causes destabilization of β‐strands located far from the binding interface. (d) Kinase GRK5 undergoes a global stabilization upon binding to β2AR. GRK5,GPCR kinase 5; HDX‐MS, hydrogen–deuterium exchange coupled to mass spectrometry; β2AR, β2 adrenergic receptor

One of the first families of IMPs to be studied by HDX‐MS was GPCRs, an interest likely sparked by their pharmacological relevance and the availability of structural data.^51^ The most thoroughly studied human GPCR is the β2 adrenergic receptor (β2AR)^52^ (Figure 3 summarizes the work carried out on β2AR). This 7‐TMs receptor transmits various external stimuli such as binding of catecholamine or synthetic drugs into intracellular signaling either by releasing the protein G complex or interacting with beta‐arrestin.^53^

#### 2.2.1 *β2 adrenergic receptor*

The first study that foresaw the value of HDX‐MS to capture the structural changes upon ligand activation dates back from 2010.^15^ This initial study focuses on the optimization of the experimental parameters and data analysis to gain adequate and reliable H/D information (Table 1). This article presents a detailed summary of the different parameters that can be optimized for increased sequence coverage, namely protein concentration, pepsin digestion and quenching conditions, chromatography parameters and peptides identification by MS. This extensive optimization led to a sequence coverage of more than 85% and provided the first comprehensive map of HDX of a folded GPCR. The authors observe that the carazolol‐bound GPCR exhibit increased H/D dynamics on TM6 compared with other helices, completely protected from exchange. A follow‐up work from the same group looked into the effect of five known ligands: agonist isoproterenol, partial agonist clenbuterol, antagonist alprenolol, and inverse agonists timolol and carazolol^54^ were all compared with the apo protein. This study established a clear difference in structural dynamics between full agonist isoproterenol and the other ligands. Specifically, an increase in HDX was observed on ICL3 and intracellular Helix‐VIII only for the full agonist (Figure 2a). In contrast, ICL2 was stabilized upon binding in all cases, except for isoproterenol. More subtle differences between antagonist alprenolol and the other ligands could also be detected, with alprenolol binding inducing an overall stronger stabilization than the other ligands, as shown by the overall reduced HDX.

Impressive work from the group of Kobilka studied the changes in structural dynamics upon formation of the active G protein‐GPCR complex.^55^ The authors performed differential HDX‐MS experiments comparing the Gs heterotrimer (Gs) alone versus in complex with β2AR, then the β2AR‐Gs complex versus the β2AR‐Gs complex trapped in a transition state with AlF^3−^ and GDP, and finally the β2AR‐Gs complex with and without GDP. In agreement with crystallographic and biochemical data, the α5 helix of the Gα subunit undergoes a significant decrease in H/D upon complex formation (Figure 3b). The most interesting feature observed by HDX‐MS is change in dynamics happening in the β1 strand. This region becomes very dynamic upon complex formation, indicating important conformational changes leading to local unfolding. It is worth noting that this region is highly conserved in Gα subunit of human G protein. This study provides a map of the structural dynamics of regions involved in mechanisms of GPCRs activation upon G protein binding.

A beautiful example of an integrative structural biology workflow uses HDX‐MS to understand the effect of β‐arrestin (βarr) recruitment by β2ar.^56^ The authors carried out differential HDX‐MS experiments where they compare the β2V2R–β‐arrestin‐1–Fab30 complex with V2Rpp–β‐arrestin‐1–Fab30 complex. HDX‐MS data show clearly the location of βarr and receptor binding, with a reduced HDX in three major loops of βarr (Figure 3c). The data suggest that such binding causes long‐range allosteric effect with local increase in dynamics of the β‐strand and the C‐domain. The authors used an integrative modeling approach that combine the HDX‐MS data with cross‐linking MS, crystallography, and disulfide trapping and modeled into a low‐resolution density EM map to generate a 3D model of the βarr‐β2ar complex.^56^

#### 2.2.2 *Frizzled 4 receptor*

Frizzled receptors are a family of GPCRs involved in Wnt signaling, and most of them bind to WnT ligands. Norrin is a cysteine‐knot growth factor, distinct from WnT ligands, that activates the canonical Wnt pathway by interacting with receptor Frizzled4 cysteine‐rich domain (Fz4_CRD_).^57^ To understand the rules of selectivity toward Norrin and the pathway for signaling, Bang et al. combined biochemical data with biophysical and computational studies.^58^ They found out that the flexible linker domain that connects the cysteine‐rich domain (CRD) to the TM domain plays an important role in signal transmission. The authors tried to capture changes in structural dynamics upon norrin binding to Fz4_CRD_ but unfortunately no peptides were detected at the interface region. Nevertheless, all the other peptides showing a change in HDX point toward the same direction: there is a global increase in structural dynamics upon Norrin binding that extends from the linker domain to the intracellular side of the TM region. The most important changes were observed on the linker domain, which corroborates the computational and biochemical findings of the study and pinpoint a role for this region in Norrin signaling.

#### 2.2.3 *C5a receptor*

Another fine example of combining HDX‐MS with structural information is found in the work of Liu et al. on the C5a receptor (C5aR).^59^ The authors solve the structure of the receptor bound to the peptide antagonist PMx53 and two different nonpeptide antagonists; avacopan or NDT9513727. The latter are located on an allosteric binding site, formed by residues on TM4, TM3, and TM5. The effect of different ligands binding on the structural dynamics of the C5aR was compared using HDX‐MS. A major difference in HDX was observed on TM7: binding of the PMx53 at the orthostetic site conferred increased protection to the cytoplasmic end of TM7, compared with the allosteric modulator NDT9513727 showing long‐range stabilizing effect on this helix.

### 2.3 *Multicomponent membrane assemblies*

Membrane proteins from less prominent drug target families were also studied by HDX‐MS, either to understand fundamental biological processes or as model systems for methodological developments. The following examples showcase the wealth of structural and dynamic information that HDX‐MS can provide to tackle more complex systems, such as multimers or multicomponent molecular machineries.

#### 2.3.1 *Microsomal glutathione transferase 1*

To the best of our knowledge, the first IMP studied by HDX‐MS was microsomal glutathione (GSH) transferase 1, a homotrimeric peroxidase that catalyzes addition of GSH to electrophilic species. In a seminal study from 2004, Busenlehner et al. used HDX‐MS to follow the conformational changes of the protein upon GSH binding and observed significant changes in HDX upon binding in several regions.^60^ The cytoplasmic loops showed protection upon binding, suggesting an overall stabilization. Interestingly, two short TM peptides showed an increase in HDX, indicating a reorientation of the trimer to allow solvent accessibility at these specific locations.

#### 2.3.2 *Cytochrome C oxidase*

The second IMP studied by HDX‐MS is cytochrome c oxidase, a redox‐driven proton pump that acts as the terminal electron acceptor in the respiratory chain of aerobic organisms. Protons are distributed between the catalytic site and exit door for pumped protons using specific proton pathways, namely the K and D pathways. The authors carried out HDX‐MS experiments on the enzyme in different oxidation states: Oxidized (O), reduced (R), “peroxy” intermediate (Pm), and “ferryl” intermediate (f). HDX‐MS showed coordinated closing and opening of the K and D proton pathway, consistent with the oxidation state of the enzyme. For example, the enzyme in the R state shows clear HDX on peptides located near the K pathway but less HDX on peptides located on the D pathway.^61^ In a follow‐up study, the authors look into the conformational effect of mutation E286H.^62^ The evolutionary conserved E286 is postulated to be the end proton acceptor of the D pathway, and responsible for shuttling protons either to the catalytic site or proton outlet. Upon mutation, the HDX‐MS profiles of the four catalytic intermediates R, O, Pm, and F are locally perturbed, showing elements of structural decoupling. Specifically, the gating loop and proton exit channel are now uncoupled, suggesting a crucial role of the acidic side chain for allosteric coordination of the different domains to allow proton passage.

#### 2.3.3 *Glycerol facilitator*

The glycerol facilitator (GF) was studied by Konermann et al. in 2012. The authors combined HDX‐MS and oxidative labeling to highlight the important role of TM7 in the structural rearrangements enabling function.^63^ The tetrameric GF protein GlpF from *Escherichia coli* facilitates passive diffusion of water and glycerol into the cytoplasm. Each monomer is made of eight TMs arranged into a pore. The selectivity for water and glycerol is puzzling and cannot be rationalized by inspection of the high‐resolution structure.^64^ The authors employed their combined approach to understand whether changes in local structural dynamics could rationalize the filtering process. They find out that the half helix TM7 exchanges unusually fast for a TM region. This observation reflects a dynamic TM region that undergoes large‐scale structural fluctuations. TM7 is part of the selectivity filter of that channel and this dynamic behavior creates a dynamic selectivity filter. This work thus suggests that the exquisite balance between fluctuation of TM7 and rigidness of the selectivity filter allows selective diffusion without jamming the pore.

#### 2.3.4 *SecA‐SecYEG complex*

**Figure 4:**
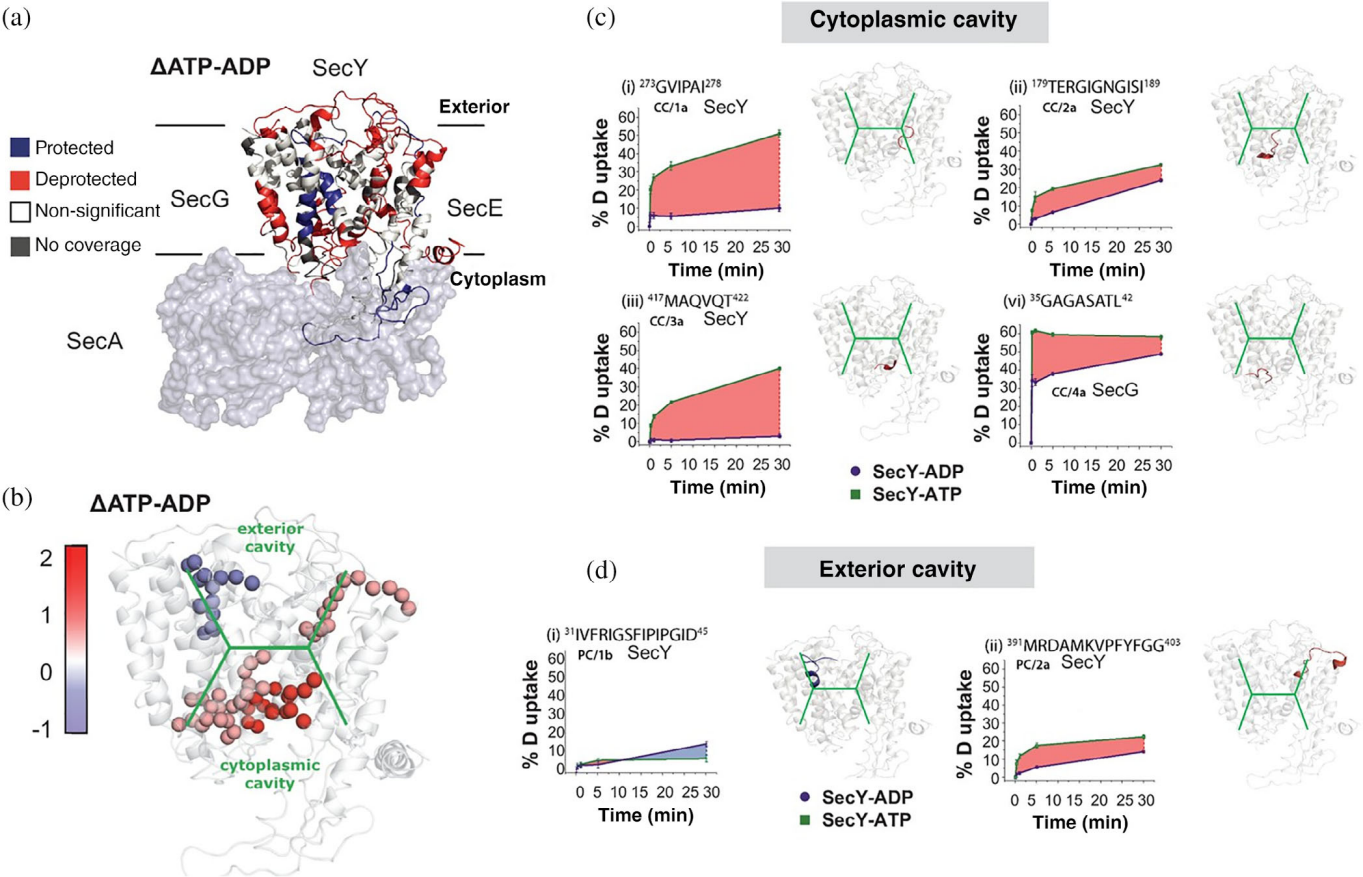
HDX data show ATP‐driven cavity changes.(a) Differences in relative deuterium uptake (ΔATP‐ADP) of SecYEG after a 30 min exposure to deuterated solvent. Blue and red colored regions indicate peptides that become HDX protected or deprotected, respectively. (b) View of SecYEG from PDB 3DIN. Shown in colored spheres are backbone nitrogens located in the SecY cavities, which exhibit a significant difference in deuterium exchange between the ATP and ADP states. The approximate positions of the respective cavities are shown in green. (c, d) Deuterium uptake plots of peptides in the SecYEG cytoplasmic cavity (CC, residues 273–278, 179–189, and 417–422 in SecY, and 35–42 in SecG) and the periplasmic cavity (PC, residues 31–45 and 391–403 in SecY). Reproduced with permission from Reference 65 (distributed under CC‐BY license). HDX, Hydrogen–deuterium exchange; PDB, Protein Data Bank coordinates

Another great example of the mechanistic insights afforded by HDX‐MS is the systematic investigation of the SecYEG‐SecA complex, involved in the translocation machinery of protein polypeptide chains. In order to understand how nucleotide binding was triggering the conformational changes enabling translocation, the authors performed ΔHDX experiments in the presence and absence of adenylyl‐imidodiphosphate (AMPPNP nonhydrolyzable analog of ATP), and ADP.^65^ This study illuminates an asymmetric allosteric change in the TM cavity of SecY (Figure 4). In a follow‐up study, the authors examine the structural dynamics of the ATPase SecA and observe that both regions are anticorrelated in terms of structural dynamics: when SecY opens SecA closes and vice versa.^66^

## 3 GOING NATIVE—BEYOND THE STATE OF THE ART

The biological membrane provides much more than a hydrophobic environment for IMPs. Both long‐range macromolecular effects and short‐range molecular interactions play an important role in the function of embedded proteins.^67^, ^68^, ^69^ For this reason, numerous membrane mimetics have been developed to combine the experimental constraints in terms of purity and concentration with an environment closer to the biological membrane.

*Bicelles* are well‐established membrane mimetics that have been extensively used for NMR studies.^70^ These planar bilayers use a specific ratio of short‐chain and long‐chain phospholipids to induce the formation of a bilayer under controlled conditions. The short‐chain phospholipid can also be replaced by select detergents such as CHAPSO.^71^ In the last 15 years, *nanodiscs* have become a very popular tool for IMPs biophysical studies. Nanodiscs allow to incorporate a purified IMP into a lipid bilayer of chosen composition surrounded by a protein belt consisting of an ApoA1 derivative.^72^ Both methods allow the formation of a bilayer around the IMP, but both start the reconstitution procedure from a detergent‐solubilized protein. Another technique that avoids the use of detergent altogether is purification with *SMA‐based polymers*.^73^ These polymers act like “cookie‐cutter” of the membrane and allow to isolate the IMP within its native lipid composition.^74^

All these different biomimetics have pros and cons and their successful application will often be protein‐dependent. They offer the advantage to interrogate the structural dynamics of IMPs within an environment matching the native one more closely. They are also used to specifically examine the role of lipid‐protein interactions. However, they bring their own share of complications for their application for HDX‐MS. The presence of lipids implies a thorough clean‐up step prior to the peptides separation which has to be quick enough to reduce back‐exchange (Table 2). Furthermore, both the digestion and the deuteration steps can be complicated by the presence of the membrane biomimetics. Different groups have, however, dedicated time and efforts to make HDX‐MS amenable to more complex and native systems. A few groups have also tried to study proteins within their native membranes, extracted from the cell, usually at the cost of much more upstream clean‐up and analysis and a bit less information (for now). The next sections review their various struggles and achievements.

**Table 2:** Sample clean‐up steps for different alternatives to detergent micelles for HDX‐MS studies

Lipid‐based systems for HDX‐MS studies of IMPs and sample clean‐up steps	
Bicelles^75^	No clean‐up step required
Nanodiscs^16^, ^18^, ^29^, ^76^	1. Addition of ZrO_2_ beads for lipid adsorption 2. Remove biotinylated MSP with neutravidin beads 3. Nanodiscs disassembly with detergent before on‐column digestion
SMALPs^77^	1. Addition of ZrO_2_ beads for lipid adsorption 2. Nanodiscs disassembly with detergent before on‐column digestion
Vesicles^78^	1. Acidification with 10% formic acid 2. Addition of soluble pepsin 3. Centrifugation and analysis of supernatant
OMVs^79^	1. Precipitation with trichloroacetic acid (TCA) 2. Wash with acetone 3. Resupension in phosphate buffer and digestion

*Note*: Left panel reviews the different systems available, going from the less to the more native systems. Right panel details the clean‐up steps devised by different research groups.

Abbreviations: HDX‐MS, hydrogen–deuterium exchange coupled to mass spectrometry; IMP, integral membrane protein; OMV, outer membrane vesicle; SMALP, Styrene Maleic Acid co‐polymer Lipid Particles

### 3.1 *Reconstitution systems: Nanodiscs and bicelles*

#### 3.1.1 *Nanodiscs*

The use of HDX‐MS to study nanodisc embedded proteins has been reviewed in detail elsewhere.^80^ This section will briefly recapitulate the different studies, major technical improvements and findings.

The first group that tackled the perilous task of performing HDX‐MS on an IMP reconstituted in nanodisc was that of John Engen. They used the human protein gamma‐glutamyl carboxylase as a model system to perform a proof‐of‐principle study in which they describe with great details the different aspects they optimized to obtain relevant structural information.^76^ The key addition to a normal HDX‐MS protocol is the use of zirconia‐coated silica beads to remove the phospholipids, as well as the addition of detergent cholate in the quench buffer to disassemble the nanodiscs (Table 2). The zirconia beads act like a Lewis base that interacts with the phosphate group of the phospholipids. Despite such optimization, the sequence coverage during HDX was on the low side (compared with detergent micelles studies), with only 51% of coverage and very few peptides from the TM regions. This benchmarking work laid the basis for a study geared toward a biological question regarding the function of the carboxylase.^81^ The effect of binding of a consensus recognition peptide was studied. The work shows unambiguously that the binding of the propeptide reduces the overall structural dynamics of the enzyme.

The next protein to be studied in nanodisc was LeuT. Two independent groups reasoned that most of the available literature on LeuT dynamics used rather invasive techniques based on the covalent modification of cysteines which can have functional consequences. H/D labeling is comparatively mild, and, furthermore, gives a global overview of the structural dynamics. Adhikary et al. reconstituted LeuT into a POPG:POPC mix. They compared the conformational dynamics of the WT with a mutant known to favor the IF conformation (see section 2.1 of Reference 18). The use of a biotinylated MSP removed with neutravidin beads before digestion, brought a significant improvement to the TM sequence coverage (Table 2). The peptide identification is greatly facilitated leading to a sequence coverage of 65%.

The ABC transporter P‐gP was also reconstituted in DMPC (Dimyristoylphosphatidylcholine) nanodiscs and studied in parallel with the protein in detergent micelles, to identify putative differences caused by the lipid environment.^29^ The coverage was not very high (37%) which limits the interpretation of the results. The authors looked for difference between the apo transporter and the vanadate‐trapped state in both environments. The protein is overall more dynamic in the apo state. The lipid environment of the nanodiscs does not seem to affect the overall pattern of HDX compared with detergent micelles. The difference between the trapped and apo states is smaller in nanodiscs, suggesting that the protein is overall less dynamic.

Another recent study took advantage of the nanodiscs technology to specifically interrogate the role of lipid‐protein interactions on the conformational ensemble. The authors reconstituted the transporters XylE and LacY in nanodiscs composed mainly of PE phospholipids (the most abundant lipid in *E. coli* bacteria) or PC lipids (absent in *E. coli*).^19^ The authors could show that the presence of PE lipids was shifting the conformational equilibrium toward the IF conformation. They corroborated these findings with MD simulations in different bilayers, which identified a specific interaction between the PE headgroup and a conserved network of charged residues shown to stabilize the OF conformation. The authors also report the best sequence coverage for proteins in nanodiscs (85%). They found that the major improvements were the use of ion mobility coupled to MS^E^ for peptide identification, adequate (~30 μM minimum) concentration of the nanodisc samples and high‐pressure (~7,000 psi) on‐line pepsin digestion (Table 2). This study laid the foundations for more systematic work on the role of specific lipid‐protein interactions on the dynamics and function of IMPs.

#### 3.1.2 *Bicelles*

In 2015, the group of Chung reconstituted three different GPCRs proteins (β2AR, μ opioid receptor, and protease activated receptor 1) in bicelles for HDX‐MS structural analysis and reported a major improvement in the sequence coverage, compared with detergent micelles^75^ (Table 2). The authors used DMPC‐CHAPSO bicelles and systemically compared coverage and redundancy for the GPCRs. They report superb coverage and redundancy for all three targets (more than 90% in all cases). The Chung group systematically used DMPC‐CHAPSO bicelles for their later studies of GPCRs. For example, they could map the binding interface of the GRk5 protein kinase when it forms a complex with the β2AR^82^ (Figure 3d). In this study, they could also observe allosteric coupling upon complex formation, manifested by a decrease in HDX in the catalytic site of the kinase. More recent work from the Chung group used pulsed HDX‐MS experiments and protein footprinting MS to dissect the sequence of events leading to assembly of the GPCR‐protein G complex.^83^ They studied assembly formation of protein G with either β2ar or A2A receptors. In both cases, the authors could identify the role of α5 helix of the Gα subunit as the starting point of complex formation. They suggest that the initial complex conformation is transient and might differ from the states captured by X‐ray crystallography and Cryo‐EM.^83^

### 3.2 *Detergent‐free HDX‐MS*

The goal of most structural biologists is to observe the molecular mechanism of their favorite membrane protein in an environment matching that of a cell as closely as possible. The use of detergent to extract proteins from the biological membrane is a step that can be detrimental, even if the protein is reinserted in a bilayer afterwards, as is the case for bicelles and nanodiscs. In that regard, the development of SMA‐based (styrene maleic acid) polymers was a welcome addition to the existing toolbox. It allows to bypass the detergent‐based solubilization step by forming so‐called “native nanodiscs,” directly solubilizing the protein with its native lipid composition preserved.

Reading et al. demonstrated the applicability of SMALPs nanodiscs for HDX‐MS analysis.^77^ The IMP rhomboid protease GlpG was expressed in different conditions (different strains of *E. coli* and growth temperatures) to alter the lipid composition of the biological membrane. The protein in three different environments were extracted from the biological membrane with SMALPs and analyzed by HDX‐MS. In parallel, the exact lipid composition of the SMALPs was determined by lipidomics. This work revealed clear differences in HDX patterns between the different lipid environments, illustrating once again the essential role of the lipids in modulating structural dynamics. Specifically, the decrease in temperature during the bacterial culture leads to increased structural dynamics in specific regions of the protease. This difference is likely caused by the increase in unsaturation in the fatty acid chains which in turn favors a more fluid bilayer.

The group of Lars Konermann did pioneering work on the Fo‐F1 ATP synthase from *E. coli*.^78^ They observed the changes in structural dynamics of the molecular motor in cation in native inverted vesicles directly extracted from *E. coli* (Table 2). The authors compared the HDX of the inhibited state bound to ADP with the active state in the presence of ATP or in the presence of ATP and a proton uncoupler carbonyl cyanide m‐chlorophenyl hydrazone (CCCP). Their most striking finding is the increased structural dynamics of the γ‐shaft of the complex in the presence of a proton‐motive force (PMF) induced by ATP hydrolysis. The authors propose that this is caused by an over‐twisting of the γ C‐helix region because of mechanical resistance of the apical bearing upon the counter‐torque exerted by the PMF.

**Figure 5:**
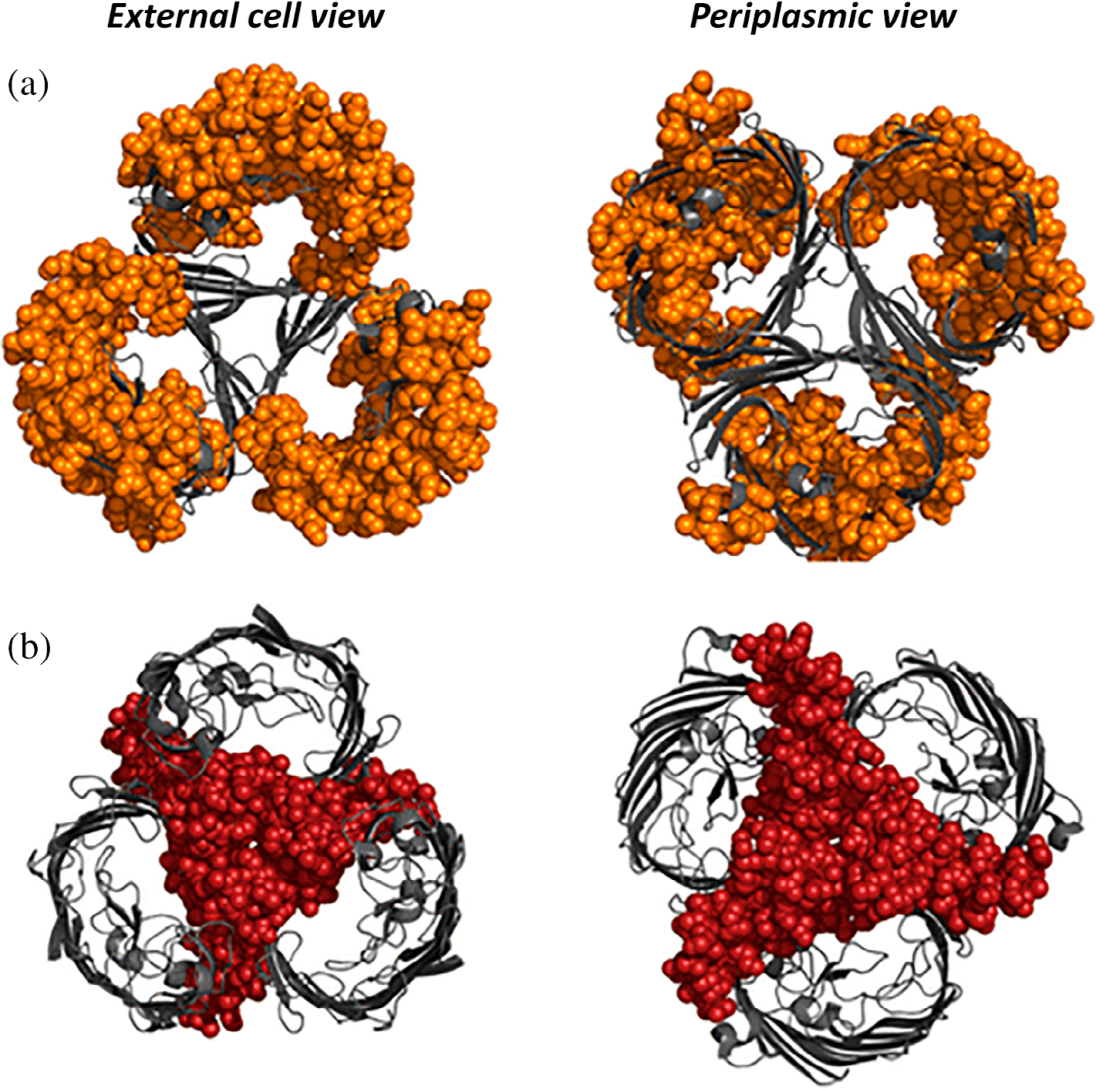
Visualization on the trimeric structure of OmpF of the peptides incorporating deuterium at slow (red) or fast (orange) kinetics. Reproduced with permission from Reference 79 (https://pubs.acs.org/doi/10.1021/acs.jproteome.7b00830). Further permissions related to the material excerpted should be directed to the ACS

Another study performed HDX‐MS experiments directly on outer membrane vesicles (OMVs) naturally released from *E. coli*.^79^ The porin OmpF is naturally over‐expressed in such membranes. This system is ideal to interrogate the in vivo organization of the porins, which has been observed both in dimeric and trimeric forms in in vitro studies. The authors devised a new strategy to get rid of the lipid contaminants of the OMV, which consists in TCA precipitation followed by acetone wash (Table 2). The authors demonstrated the validity of their methodology by showing that the HDX pattern followed one expected from the in vivo trimeric organization of the porins (Figure 5).

## 4 CONCLUDING REMARKS

This review provides an inventory of the work that has been done on IMPs by HDX‐MS. The evident conclusion is that the wealth of information afforded by the technique is directly proportional to the level of a priori structural and biochemical knowledge of the system under investigation. In that regard, HDX‐MS is ideally positioned to provide unique molecular insights into mechanistical questions and is likely to be used in a more systematic way in more structural biology studies. As integrative approaches combining biophysical, structural and biochemical data are more and more required to understand fundamental biological mechanisms, HDX‐MS is certain to shine in the set of biophysical tools accompanying the resolution revolution. A recent study on the Cop9 Signalosome—CRL2 supercomplex provided early evidence of how Cryo‐EM and HDX‐MS can go hand to hand for deciphering mechanistic insights otherwise inaccessible by each technique in isolation.^84^

The ability to look into complex and heterogeneous systems and extracting a signal unique to the protein of interest (at a peptide level of resolution) without invasive labeling and restriction in terms of size is an unmatched advantage. Improvement of automation and data analysis, peptide assignment, good practice, will hopefully facilitate the widespread use of this technique.

## AUTHOR CONTRIBUTIONS

**Chloe Martens:** Conceptualization; investigation; writing‐original draft; writing‐review and editing. **Argyris Politis:** Conceptualization; funding acquisition; investigation; writing‐original draft; writing‐review and editing.

## References

[1] Seddon AM , Curnow P , Booth PJ . Membrane proteins, lipids and detergents: Not just a soap opera. Biochim Biophys Acta. 2004;1666:105–117. 15519311 10.1016/j.bbamem.2004.04.011

[2] Trabjerg E , Nazari ZE , Rand KD . Conformational analysis of complex protein states by hydrogen/deuterium exchange mass spectrometry (HDX‐MS): Challenges and emerging solutions. Trends in Analyt Chem. 2018;106:125–138.

[3] Masson GR , Jenkins ML , Burke JE . An overview of hydrogen deuterium exchange mass spectrometry (HDX‐MS) in drug discovery. Expert Opin Drug Discov. 2017;12:981–994. 28770632 10.1080/17460441.2017.1363734

[4] Engen JR . Analysis of protein conformation and dynamics by hydrogen/deuterium exchange MS. Anal Chem. 2009;81:7870–7875. 19788312 10.1021/ac901154s PMC2756691

[5] Faini M , Stengel F , Aebersold R . The evolving contribution of mass spectrometry to integrative structural biology. J Am Soc Mass Spectrom. 2016;27:966–974. 27056566 10.1007/s13361-016-1382-4 PMC4867889

[6] Oganesyan I , Lento C , Wilson DJ . Contemporary hydrogen deuterium exchange mass spectrometry. Methods. 2018;144:27–42. 29704663 10.1016/j.ymeth.2018.04.023

[7] Hodge EA , Benhaim MA , Lee KK . Bridging protein structure, dynamics, and function using hydrogen/deuterium‐exchange mass spectrometry. Protein Sci. 2019 10.1002/pro.3790. PMC7096709 31721348

[8] Weis DD . Hydrogen exchange mass spectrometry of proteins: Fundamentals, methods, and applications. West Sussex, UK: John Wiley & Sons, Ltd., 2016.

[9] Rey M , Man P , Brandolin G , Forest E , Pelosi L . Recombinant immobilized rhizopuspepsin as a new tool for protein digestion in hydrogen/deuterium exchange mass spectrometry. Rapid Commun Mass Spectrom. 2009;23:3431–3438. 19827048 10.1002/rcm.4260

[10] Rey M , Yang M , Burns KM , Yu Y , Lees‐Miller SP , Schriemer DC . Nepenthesin from monkey cups for hydrogen/deuterium exchange mass spectrometry. Mol Cell Proteomics. 2013;12:464–472. 23197791 10.1074/mcp.M112.025221 PMC3567866

[11] Moller IR , Slivacka M , Hausner J , et al. Improving the sequence coverage of integral membrane proteins during hydrogen/deuterium exchange mass spectrometry experiments. Anal Chem. 2019;91:10970–10978. 31408320 10.1021/acs.analchem.9b00973

[12] Wang L , Pan H , Smith DL . Hydrogen exchange‐mass spectrometry: Optimization of digestion conditions. Mol Cell Proteomics. 2002;1:132–138. 12096131 10.1074/mcp.m100009-mcp200

[13] Kopcho N , Chang G , Komives EA . Dynamics of ABC transporter P‐glycoprotein in three conformational states. Sci Rep. 2019;9:15092. 31641149 10.1038/s41598-019-50578-2 PMC6805939

[14] Harris MJ , Raghavan D , Borysik AJ . Quantitative evaluation of native protein folds and assemblies by hydrogen deuterium exchange mass spectrometry (HDX‐MS). J Am Soc Mass Spectrom. 2019;30:58–66. 30280315 10.1007/s13361-018-2070-3 PMC6318237

[15] Zhang X , Chien EY , Chalmers MJ , et al. Dynamics of the beta2‐adrenergic G‐protein coupled receptor revealed by hydrogen‐deuterium exchange. Anal Chem. 2010;82:1100–1108. 20058880 10.1021/ac902484p PMC2829980

[16] Martens C , Shekhar M , Lau AM , Tajkhorshid E , Politis A . Integrating hydrogen‐deuterium exchange mass spectrometry with molecular dynamics simulations to probe lipid‐modulated conformational changes in membrane proteins. Nat Protoc. 2019;14:3183–3204. 31605097 10.1038/s41596-019-0219-6 PMC7058097

[17] Distler U , Kuharev J , Navarro P , Levin Y , Schild H , Tenzer S . Drift time‐specific collision energies enable deep‐coverage data‐independent acquisition proteomics. Nat Methods. 2014;11:167–170. 24336358 10.1038/nmeth.2767

[18] Adhikary S , Deredge DJ , Nagarajan A , Forrest LR , Wintrode PL , Singh SK . Conformational dynamics of a neurotransmitter: Sodium symporter in a lipid bilayer. Proc Natl Acad Sci U S A. 2017;114:E1786–E1795. 28223522 10.1073/pnas.1613293114 PMC5347597

[19] Martens C , Shekhar M , Borysik AJ , et al. Direct protein‐lipid interactions shape the conformational landscape of secondary transporters. Nat Commun. 2018;9:4151. 30297844 10.1038/s41467-018-06704-1 PMC6175955

[20] McAllister RG , Konermann L . Challenges in the interpretation of protein h/d exchange data: A molecular dynamics simulation perspective. Biochemistry. 2015;54:2683–2692. 25860179 10.1021/acs.biochem.5b00215

[21] Weis DD . Comment on Houde D, Berkowitz SA, Engen JR. The utility of hydrogen/deuterium exchange mass spectrometry in biopharmaceutical comparability studies. J Pharm Sci (2011) 100:2071‐2086. J Pharm Sci. 2019;108:807–810. 30339865 10.1016/j.xphs.2018.10.010 PMC6351210

[22] Fang J , Rand KD , Beuning PJ , Engen JR . False EX1 signatures caused by sample carryover during HX MS analyses. Int J Mass Spectrom. 2011;302:19–25. 21643454 10.1016/j.ijms.2010.06.039 PMC3106990

[23] Masson GR , Burke JE , Ahn NG , et al. Recommendations for performing, interpreting and reporting hydrogen deuterium exchange mass spectrometry (HDX‐MS) experiments. Nat Methods. 2019;16:595–602. 31249422 10.1038/s41592-019-0459-y PMC6614034

[24] Jardetzky O . Simple allosteric model for membrane pumps. Nature. 1966;211:969–970. 5968307 10.1038/211969a0

[25] Ryan RM , Vandenberg RJ . Elevating the alternating‐access model. Nat Struct Mol Biol. 2016;23:187–189. 26931415 10.1038/nsmb.3179

[26] Slotboom DJ . Structural and mechanistic insights into prokaryotic energy‐coupling factor transporters. Nat Rev Microbiol. 2014;12:79–87. 24362466 10.1038/nrmicro3175

[27] Mehmood S , Domene C , Forest E , Jault JM . Dynamics of a bacterial multidrug ABC transporter in the inward‐ and outward‐facing conformations. Proc Natl Acad Sci U S A. 2012;109:10832–10836. 22711831 10.1073/pnas.1204067109 PMC3390859

[28] Orelle C , Dalmas O , Gros P , Di Pietro A , Jault JM . The conserved glutamate residue adjacent to the Walker‐B motif is the catalytic base for ATP hydrolysis in the ATP‐binding cassette transporter BmrA. J Biol Chem. 2003;278:47002–47008. 12968023 10.1074/jbc.M308268200

[29] Li MJ , Guttman M , Atkins WM . Conformational dynamics of P‐glycoprotein in lipid nanodiscs and detergent micelles reveal complex motions on a wide time scale. J Biol Chem. 2018;293:6297–6307. 29511086 10.1074/jbc.RA118.002190 PMC5925813

[30] Chen Z , Shi T , Zhang L , et al. Mammalian drug efflux transporters of the ATP binding cassette (ABC) family in multidrug resistance: A review of the past decade. Cancer Lett. 2016;370:153–164. 26499806 10.1016/j.canlet.2015.10.010

[31] Dastvan R , Mishra S , Peskova YB , Nakamoto RK , McHaourab HS . Mechanism of allosteric modulation of P‐glycoprotein by transport substrates and inhibitors. Science. 2019;364:689–692. 31097669 10.1126/science.aav9406 PMC6890515

[32] Szollosi D , Rose‐Sperling D , Hellmich UA , Stockner T . Comparison of mechanistic transport cycle models of ABC exporters. Biochim Biophys Acta Biomembr. 2018;1860:818–832. 29097275 10.1016/j.bbamem.2017.10.028 PMC7610611

[33] Penmatsa A , Gouaux E . How LeuT shapes our understanding of the mechanisms of sodium‐coupled neurotransmitter transporters. J Physiol. 2014;592:863–869. 23878376 10.1113/jphysiol.2013.259051 PMC3948551

[34] Kanner BI , Zomot E . Sodium‐coupled neurotransmitter transporters. Chem Rev. 2008;108:1654–1668. 18393466 10.1021/cr078246a

[35] Penmatsa A , Wang KH , Gouaux E . X‐ray structure of dopamine transporter elucidates antidepressant mechanism. Nature. 2013;503:85–90. 24037379 10.1038/nature12533 PMC3904663

[36] Coleman JA , Green EM , Gouaux E . X‐ray structures and mechanism of the human serotonin transporter. Nature. 2016;532:334–339. 27049939 10.1038/nature17629 PMC4898786

[37] Merkle PS , Gotfryd K , Cuendet MA , et al. Substrate‐modulated unwinding of transmembrane helices in the NSS transporter LeuT. Sci Adv. 2018;4:eaar6179. 29756037 10.1126/sciadv.aar6179 PMC5947982

[38] Krishnamurthy H , Gouaux E . X‐ray structures of LeuT in substrate‐free outward‐open and apo inward‐open states. Nature. 2012;481:469–474. 22230955 10.1038/nature10737 PMC3306218

[39] Nielsen AK , Moller IR , Wang Y , et al. Substrate‐induced conformational dynamics of the dopamine transporter. Nat Commun. 2019;10:2714. 31221956 10.1038/s41467-019-10449-w PMC6586795

[40] Moller IR , Slivacka M , Nielsen AK , et al. Conformational dynamics of the human serotonin transporter during substrate and drug binding. Nat Commun. 2019;10:1687. 30976000 10.1038/s41467-019-09675-z PMC6459873

[41] Saier MH Jr , Beatty JT , Goffeau A , et al. The major facilitator superfamily. J Mol Microbiol Biotechnol. 1999;1:257–279. 10943556

[42] Rey M , Man P , Clemencon B , et al. Conformational dynamics of the bovine mitochondrial ADP/ATP carrier isoform 1 revealed by hydrogen/deuterium exchange coupled to mass spectrometry. J Biol Chem. 2010;285:34981–34990. 20805227 10.1074/jbc.M110.146209 PMC2966112

[43] Ruprecht JJ , King MS , Zogg T , et al. The molecular mechanism of transport by the mitochondrial ADP/ATP varrier. Cell. 2019;176:435–447. 30611538 10.1016/j.cell.2018.11.025 PMC6349463

[44] Clemencon B , Rey M , Trezeguet V , Forest E , Pelosi L . Yeast ADP/ATP carrier isoform 2 conformational dynamics and role of the RRRMMM signature sequence methionines. J Biol Chem. 2011;286:36119–36131. 21868387 10.1074/jbc.M111.277376 PMC3195597

[45] Canul‐Tec JC , Assal R , Cirri E , et al. Structure and allosteric inhibition of excitatory amino acid transporter 1. Nature. 2017;544:446–451. 28424515 10.1038/nature22064 PMC5410168

[46] Eisinger ML , Dorrbaum AR , Michel H , Padan E , Langer JD . Ligand‐induced conformational dynamics of the *Escherichia coli* Na(+)/H(+) antiporter NhaA revealed by hydrogen/deuterium exchange mass spectrometry. Proc Natl Acad Sci U S A. 2017;114:11691–11696. 29078272 10.1073/pnas.1703422114 PMC5676877

[47] Giladi M , van Dijk L , Refaeli B , et al. Dynamic distinctions in the Na(+)/Ca(2+) exchanger adopting the inward‐ and outward‐facing conformational states. J Biol Chem. 2017;292:12311–12323. 28572509 10.1074/jbc.M117.787168 PMC5519378

[48] Liao J , Li H , Zeng W , Sauer DB , Belmares R , Jiang Y . Structural insight into the ion‐exchange mechanism of the sodium/calcium exchanger. Science. 2012;335:686–690. 22323814 10.1126/science.1215759

[49] Marinelli F , Almagor L , Hiller R , Giladi M , Khananshvili D , Faraldo‐Gomez JD . Sodium recognition by the Na+/Ca2+ exchanger in the outward‐facing conformation. Proc Natl Acad Sci U S A. 2014;111:E5354–E5362. 25468964 10.1073/pnas.1415751111 PMC4273333

[50] Eisinger ML , Nie L , Dorrbaum AR , Langer JD , Michel H . The xenobiotic extrusion mechanism of the MATE transporter NorM_PS from *Pseudomonas stutzeri* . J Mol Biol. 2018;430:1311–1323. 29555555 10.1016/j.jmb.2018.03.012

[51] Congreve M , Marshall F . The impact of GPCR structures on pharmacology and structure‐based drug design. Br J Pharmacol. 2010;159:986–996. 19912230 10.1111/j.1476-5381.2009.00476.x PMC2839258

[52] Rasmussen SG , Choi HJ , Rosenbaum DM , et al. Crystal structure of the human beta2 adrenergic G‐protein‐coupled receptor. Nature. 2007;450:383–387. 17952055 10.1038/nature06325

[53] Hilger D , Masureel M , Kobilka BK . Structure and dynamics of GPCR signaling complexes. Nat Struct Mol Biol. 2018;25:4–12. 29323277 10.1038/s41594-017-0011-7 PMC6535338

[54] West GM , Chien EY , Katritch V , et al. Ligand‐dependent perturbation of the conformational ensemble for the GPCR beta2 adrenergic receptor revealed by HDX. Structure. 2011;19:1424–1432. 21889352 10.1016/j.str.2011.08.001 PMC3196059

[55] Chung KY , Rasmussen SG , Liu T , et al. Conformational changes in the G protein Gs induced by the beta2 adrenergic receptor. Nature. 2011;477:611–615. 21956331 10.1038/nature10488 PMC3448949

[56] Shukla AK , Westfield GH , Xiao K , et al. Visualization of arrestin recruitment by a G‐protein‐coupled receptor. Nature. 2014;512:218–222. 25043026 10.1038/nature13430 PMC4134437

[57] Ye X , Wang Y , Nathans J . The Norrin/Frizzled4 signaling pathway in retinal vascular development and disease. Trends Mol Med. 2010;16:417–425. 20688566 10.1016/j.molmed.2010.07.003 PMC2963063

[58] Bang I , Kim HR , Beaven AH , et al. Biophysical and functional characterization of Norrin signaling through Frizzled4. Proc Natl Acad Sci U S A. 2018;115:8787–8792. 30104375 10.1073/pnas.1805901115 PMC6126767

[59] Liu H , Kim HR , Deepak RNVK , et al. Orthosteric and allosteric action of the C5a receptor antagonists. Nat Struct Mol Biol. 2018;25:472–481. 29867214 10.1038/s41594-018-0067-z

[60] Busenlehner LS , Codreanu SG , Holm PJ , et al. Stress sensor triggers conformational response of the integral membrane protein microsomal glutathione transferase 1. Biochemistry. 2004;43:11145–11152. 15366924 10.1021/bi048716k

[61] Busenlehner LS , Salomonsson L , Brzezinski P , Armstrong RN . Mapping protein dynamics in catalytic intermediates of the redox‐driven proton pump cytochrome c oxidase. Proc Natl Acad Sci U S A. 2006;103:15398–15403. 17023543 10.1073/pnas.0601451103 PMC1622835

[62] Busenlehner LS , Branden G , Namslauer I , Brzezinski P , Armstrong RN . Structural elements involved in proton translocation by cytochrome c oxidase as revealed by backbone amide hydrogen‐deuterium exchange of the E286H mutant. Biochemistry. 2008;47:73–83. 18052347 10.1021/bi701643a

[63] Pan Y , Piyadasa H , O'Neil JD , Konermann L . Conformational dynamics of a membrane transport protein probed by H/D exchange and covalent labeling: The glycerol facilitator. J Mol Biol. 2012;416:400–413. 22227391 10.1016/j.jmb.2011.12.052

[64] Fu D , Libson A , Miercke LJ , et al. Structure of a glycerol‐conducting channel and the basis for its selectivity. Science. 2000;290:481–486. 11039922 10.1126/science.290.5491.481

[65] Corey RA , Ahdash Z , Shah A , et al. ATP‐induced asymmetric pre‐protein folding as a driver of protein translocation through the Sec machinery. Elife. 2019;8:e41803. 30601115 10.7554/eLife.41803 PMC6335059

[66] Ahdash Z , Pyle E , Allen WJ , Corey RA , Collinson I , Politis A . HDX‐MS reveals nucleotide‐dependent, anti‐correlated opening and closure of SecA and SecY channels of the bacterial translocon. Elife. 2019;8:e47402. 31290743 10.7554/eLife.47402 PMC6639072

[67] Laganowsky A , Reading E , Allison TM , et al. Membrane proteins bind lipids selectively to modulate their structure and function. Nature. 2014;510:172–175. 24899312 10.1038/nature13419 PMC4087533

[68] Lee AG . How lipids affect the activities of integral membrane proteins. Biochim Biophys Acta. 2004;1666:62–87. 15519309 10.1016/j.bbamem.2004.05.012

[69] Martens C , Stein RA , Masureel M , et al. Lipids modulate the conformational dynamics of a secondary multidrug transporter. Nat Struct Mol Biol. 2016;23:744–751. 27399258 10.1038/nsmb.3262 PMC5248563

[70] Sanders CR II , Landis GC . Reconstitution of membrane proteins into lipid‐rich bilayered mixed micelles for NMR studies. Biochemistry. 1995;34:4030–4040. 7696269 10.1021/bi00012a022

[71] Czerski L , Sanders CR . Functionality of a membrane protein in bicelles. Anal Biochem. 2000;284:327–333. 10964416 10.1006/abio.2000.4720

[72] Bayburt TH , Sligar SG . Membrane protein assembly into nanodiscs. FEBS Lett. 2010;584:1721–1727. 19836392 10.1016/j.febslet.2009.10.024 PMC4758813

[73] Knowles TJ , Finka R , Smith C , Lin YP , Dafforn T , Overduin M . Membrane proteins solubilized intact in lipid containing nanoparticles bounded by styrene maleic acid copolymer. J Am Chem Soc. 2009;131:7484–7485. 19449872 10.1021/ja810046q

[74] Lee SC , Knowles TJ , Postis VL , et al. A method for detergent‐free isolation of membrane proteins in their local lipid environment. Nat Protoc. 2016;11:1149–1162. 27254461 10.1038/nprot.2016.070

[75] Duc NM , Du Y , Zhang C , et al. Effective application of bicelles for conformational analysis of G protein‐coupled receptors by hydrogen/deuterium exchange mass spectrometry. J Am Soc Mass Spectrom. 2015;26:808–817. 25740347 10.1007/s13361-015-1083-4 PMC4727453

[76] Hebling CM , Morgan CR , Stafford DW , Jorgenson JW , Rand KD , Engen JR . Conformational analysis of membrane proteins in phospholipid bilayer nanodiscs by hydrogen exchange mass spectrometry. Anal Chem. 2010;82:5415–5419. 20518534 10.1021/ac100962c PMC2895417

[77] Reading E , Hall Z , Martens C , et al. Interrogating membrane protein conformational dynamics within native lipid compositions. Angew Chem Int Ed Engl. 2017;56:15654–15657. 29049865 10.1002/anie.201709657

[78] Vahidi S , Bi Y , Dunn SD , Konermann L . Load‐dependent destabilization of the gamma‐rotor shaft in FOF1 ATP synthase revealed by hydrogen/deuterium‐exchange mass spectrometry. Proc Natl Acad Sci U S A. 2016;113:2412–2417. 26884184 10.1073/pnas.1520464113 PMC4780623

[79] Donnarumma D , Maestri C , Giammarinaro PI , et al. Native state organization of outer membrane porins unraveled by HDx‐MS. J Proteome Res. 2018;17:1794–1800. 29619829 10.1021/acs.jproteome.7b00830

[80] Redhair M , Clouser AF , Atkins WM . Hydrogen‐deuterium exchange mass spectrometry of membrane proteins in lipid nanodiscs. Chem Phys Lipids. 2019;220:14–22. 30802434 10.1016/j.chemphyslip.2019.02.007 PMC6480397

[81] Parker CH , Morgan CR , Rand KD , Engen JR , Jorgenson JW , Stafford DW . A conformational investigation of propeptide binding to the integral membrane protein gamma‐glutamyl carboxylase using nanodisc hydrogen exchange mass spectrometry. Biochemistry. 2014;53:1511–1520. 24512177 10.1021/bi401536m PMC3970815

[82] Komolov KE , Du Y , Duc NM , et al. Structural and functional analysis of a beta2‐adrenergic receptor complex with GRK5. Cell. 2017;169:407–421. 28431242 10.1016/j.cell.2017.03.047 PMC5526774

[83] Du Y , Duc NM , Rasmussen SGF , et al. Assembly of a GPCR‐G protein complex. Cell. 2019;177:1232–1242. 31080064 10.1016/j.cell.2019.04.022 PMC6763313

[84] Faull SV , Lau AMC , Martens C , et al. Structural basis of Cullin 2 RING E3 ligase regulation by the COP9 signalosome. Nat Commun. 2019;10:3814. 31444342 10.1038/s41467-019-11772-y PMC6707232

